# Pleiotropic immunoregulation by bile acids in pathophysiology

**DOI:** 10.3389/fimmu.2026.1719092

**Published:** 2026-02-05

**Authors:** Bo-Shen Lin, Min-Yue Yin, Si-An Xie, Peng Li, Xue Li

**Affiliations:** 1 Department of Gastroenterology, Beijing Friendship Hospital, Capital Medical University, State Key Laboratory of Digestive Health, National Clinical Research Center for Digestive Disease, Beijing Key Laboratory of Early Gastrointestinal Cancer Medicine and Medical Devices, Beijing, China; 2 Department of Gastroenterology, Sichuan Provincial People’s Hospital, School of Medicine, University of Electronic Science and Technology of China, Chengdu, China

**Keywords:** autoimmune diseases, bile acids, cancer, gut microbiota, immunity

## Abstract

Bile acids (BAs) have evolved from their classical role in lipid digestion to become central signaling molecules that integrate host metabolism, gut microbiota, and immune function. This review examines how diverse BAs regulate both innate and adaptive immunity through specific receptors—including farnesoid X receptor, Takeda G-protein-coupled receptor 5, vitamin D receptor, and retinoid orphan receptors—modulating the activity of macrophages, dendritic cells, T cells, natural killer cells, and natural killer T cells. Tissue−specific BA signaling influences immune homeostasis in the intestine, liver, central nervous system, and tumor microenvironment. Furthermore, we discuss the pathogenic role of dysregulated BA signaling in inflammatory, autoimmune, metabolic, and malignant diseases, and evaluate emerging therapeutic strategies that target BA pathways via synthetic ligands, engineered microbes, and dietary modulation. Leveraging BA-immune crosstalk to advance research on precision immunotherapy and microbiome-based interventions is a promising area of research.

## 1 Introduction

**Figure 1:**
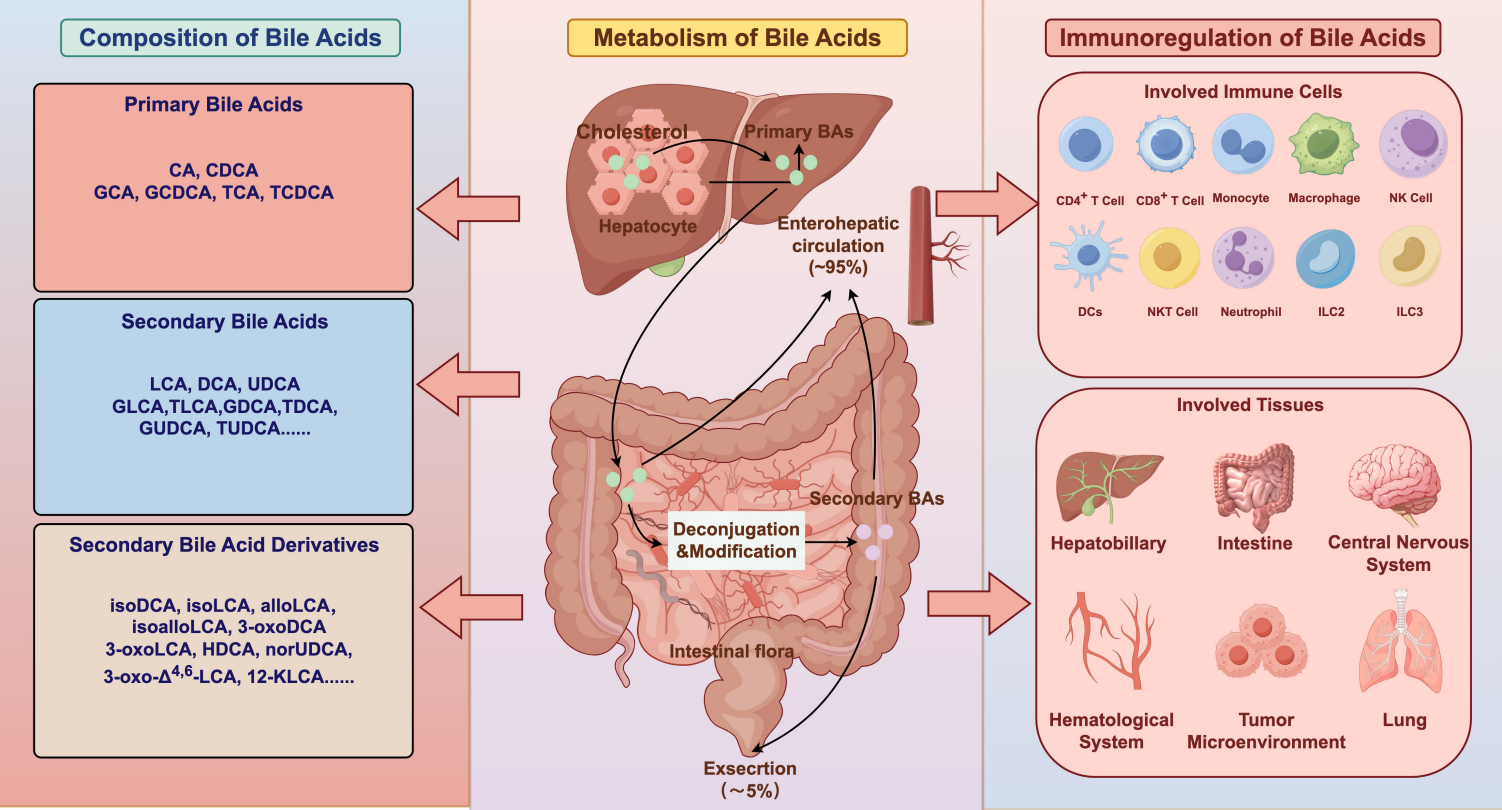
Bile acid composition, metabolism, and immunoregulatory functions. Primary bile acids produced in the liver are transformed by gut microbial metabolism into a broad spectrum of secondary bile acids and derivatives and undergo extensive enterohepatic circulation. Beyond their metabolic roles, bile acids function as signaling molecules that modulate diverse immune cell populations, thereby shaping immune homeostasis and inflammatory responses across multiple tissues. BAs, Bile Acids; CA, Cholic Acid; CDCA, Chenodeoxycholic Acid; GCA, Glycocholic Acid; TCA, Taurocholic Acid; TCDCA, Taurochenodeoxycholic Acid; GCDCA, Glycochenodeoxycholic Acid; LCA, Lithocholic Acid; DCA, Deoxycholic Acid; UDCA, Ursodeoxycholic Acid; GLCA, Glycolithocholic Acid; TLCA, Taurolithocholic Acid; TDCA, Taurodeoxycholic Acid; GDCA, Glycodeoxycholic Acid; TUDCA, Tauroursodeoxycholic Acid; GUDCA, Glycoursodeoxycholic Acid; isoDCA, 3β-hydroxydeoxycholic acid; isoLCA, Isolithocholic Acid; alloLCA, allo−lithocholic Acid; isoalloLCA, isoallolithocholic Acid; 3-oxoDCA, 3-oxodeoxycholic Acid; 3-oxoLCA, 3-oxolithocholic Acid; HDCA, Hyodeoxycholic Acid; norUDCA, 24-Nor-ursodeoxycolic Acid; 3-oxo-Δ^4,6^-LCA, 3-oxo-Δ^4,6^-lithocholic Acid; 12-KLCA, 12-ketolithocholic Acids; NK, Natural Killer; ILC3: Group 3 Innate Lymphoid Cell; ILC2: Group 2 Innate Lymphoid Cell; DCs, Dendritic cells; NKT, Natural Killer T.

Primary bile acids (BAs) are predominantly synthesized within hepatocytes and secreted into the intestinal lumen, where they serve as essential agents for lipid digestion and absorption (1). While a small proportion of BAs are eliminated via fecal or urinary excretion, the majority are reabsorbed and returned to the liver through the enterohepatic circulation (2). In the liver, primary BAs, such as cholic acid (CA) and chenodeoxycholic acid (CDCA), are typically conjugated with glycine or taurine to form conjugated BAs. Upon reaching the intestine, these BAs undergo extensive structural transformation by gut microbiota enzymes, generating secondary BAs, including lithocholic acid (LCA) and deoxycholic acid (DCA), as well as structurally related isomers such as isolithocholic acid (isoLCA), 3−oxolithocholic acid (3−oxoLCA), 3−oxoallo−lithocholic acid (3−oxoalloLCA), allo−lithocholic acid (alloLCA), and isoallolithocholic acid (isoalloLCA) (**Figure 1**). Advances in high-resolution mass spectrometry coupled with computational tools have expanded the BA repertoire, enabling the in silico identification and classification of nearly 200 previously unrecognized BA species (3).

Besides their canonical digestive functions, BAs are key signaling molecules that regulate a wide range of physiological processes, including immunomodulation (4). Different BA species can activate or suppress specific immune cell populations, influencing the onset and progression of various inflammatory diseases and cancer (5). This review elucidates the mechanisms by which distinct BAs modulate immune cell functions and downstream signaling pathways, and discusses their emerging therapeutic relevance in disease contexts.

## 2 Receptor-specific control of immune responses by BAs

Since the identification of the farnesoid X receptor (FXR) as the first BA receptor, BAs have been recognized as vital signaling molecules (6, 7). Although numerous BA receptors have been identified, those implicated in BA-mediated immune regulation are limited to the FXR, Takeda G protein-coupled receptor 5 (TGR5), vitamin D receptor (VDR), retinoid orphan receptors (RORs), nuclear receptor subfamily 4 group A member 1 (NR4A1), constitutive androstane receptor (CAR), human androgen receptor (hAR) (8–11). Emerging evidence indicates that BAs also modulate immune cell function through specific receptors, shaping systemic immune responses and contributing to diseases such as inflammation and tumorigenesis (12). These pathways collectively shape myeloid activation, epithelial tolerance, and the Th17-Treg balance, linking microbial BA metabolism to host inflammatory responses and underscoring their substantial scientific value in decoding immunometabolic regulation and host-microbiota crosstalk.

### 2.1 FXR

FXR, a key member of the nuclear receptor superfamily, functions as a central transcription factor and is highly expressed in the liver and intestines (6, 13, 14). FXR is also present in various myeloid and innate immune cells, including monocytes, macrophages, dendritic cells (DCs), natural killer (NK) cells, and natural killer T (NKT) cells, although its expression in T cells is relatively low (15). Several endogenous BAs act as FXR agonists, including CA, CDCA, LCA, DCA, taurocholic acid (TCA), and synthetic derivative 6-ethylchenodeoxycholic acid (6E-CDCA) (16–18). Conversely, specific BA species, such as 3β-hydroxydeoxycholic acid (isoDCA), 7-ketolithocholic acid (7-ketoLCA), and α/β-muricholic acid (MCA), serve as antagonists of FXR (19–21). Ursodeoxycholic acid (UDCA) exhibits concentration-dependent dual activity and can serve as either an agonist or antagonist of FXR, highlighting the complexity of BA-receptor interactions (20, 22).

**Figure 2:**
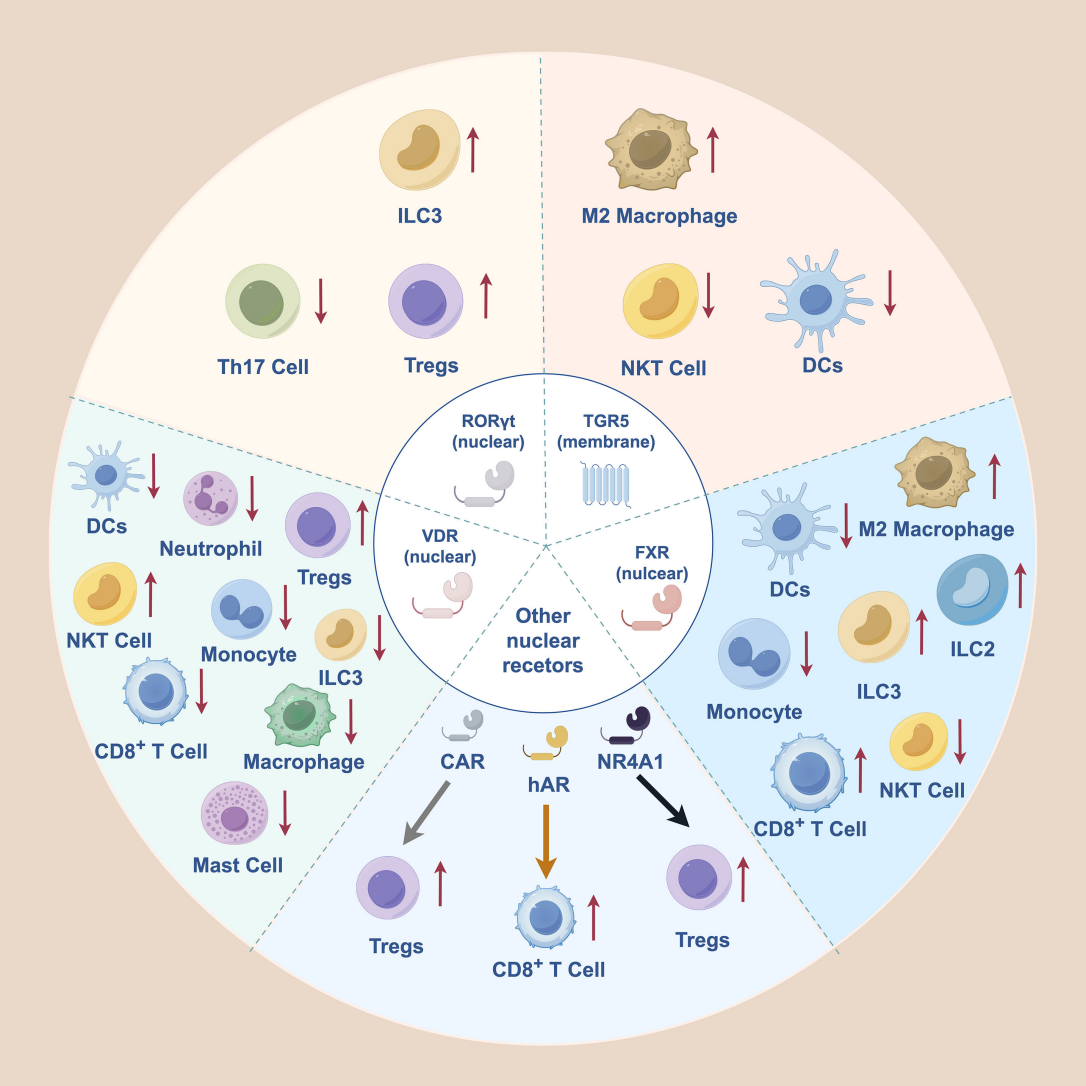
Receptor-specific immunomodulatory effects of bile acids on innate and adaptive immune cells. Distinct bile acid receptors, including the nuclear receptors FXR, VDR, RORγt, CAR, NR4A1, and hAR, as well as the membrane receptor TGR5, mediate bile acid signaling in diverse immune cell populations. Through these pathways, bile acids differentially modulate immune cell activation, differentiation, and functional polarization, influencing Treg-Th17 balance, macrophage polarization, dendritic cell activity, innate lymphoid cell subsets, and cytotoxic lymphocyte responses. The directionality of regulation (upregulation or suppression) varies by receptor context and cell type, highlighting the pleiotropic and context-dependent roles of bile acids in shaping immune homeostasis and inflammatory outcomes. FXR, Farnesoid X Receptor; TGR5, Takeda G protein-coupled receptor 5; VDR, Vitamin D Receptor; RORγT, Retinoic Acid Receptor-related Orphan Receptor-γT; NR4A1, Orphan Nuclear Receptor 4A1; CAR, Constitutive Androstane Receptor; hAR, human Androgen Receptor; ILC3: Group 3 Innate Lymphoid Cell; ILC2: Group 2 Innate Lymphoid Cell; DCs, Dendritic cells, NKT, natural killer T; Tregs, regulatory T cells; Th17, T helper 17 cells.

FXR plays a multifaceted role in regulating innate immune cells, particularly macrophages and DCs, to maintain immune homeostasis (**Figure 2**). In macrophages, FXR activation by agonists such as 6E-CDCA or UDCA suppresses pro-inflammatory signaling by stabilizing the NCoR-NF−κB complex, which reduces cytokine release such as IL−1β, TNF−α, IL−6, and inducible nitric oxide synthase (iNOS). It also directly disrupts NOD-like receptor pyrin domain containing 3 (NLRP3) inflammasome assembly, preventing caspase−1−dependent maturation of IL−1β (18, 23). Furthermore, FXR promotes a shift toward the anti−inflammatory M2 phenotype, partly via retinoic acid signaling (24). In DCs, FXR activation impairs monocyte−to−DC differentiation and reduces DC activation, as evidenced by 6E−CDCA−mediated suppression of DC maturation *in vitro* and reduced colonic DC activation in dextran sulphate sodium (DSS)−colitis models (25, 26). Conversely, FXR antagonism by isoDCA diminishes DC immunostimulatory capacity and fosters a tolerogenic microenvironment that promotes peripheral regulatory T cell (Treg) differentiation (19). In ILC2, CA and CDCA-induced activation via FXR promoted IL-33 expression, thereby accelerating intestinal inflammation (27).

FXR also significantly influences adaptive immune responses. It increases the expression of its target gene, small heterodimer partner (SHP), which enhances SHP’s interaction with c-Jun. This interaction prevented c-Jun from binding to the OPN promoter, leading to a decrease in OPN production in NKT cells. Therefore, NKT cell activation and function are suppressed, ultimately reducing immune-mediated liver damage (28). Under conditions of nutrient scarcity during infection, FXR is upregulated in activated CD8^+^ T cells, which limits the utilization of alternative fuels, such as glutamine and fatty acids, reducing metabolic flexibility and promoting contraction of the effector T-cell pool to maintain host energy homeostasis (29). Collectively, these findings highlight FXR as a key immunoregulatory node in innate and adaptive immune cells, and its targeted modulation may offer novel strategies for enhancing immunotherapeutic outcomes in inflammatory diseases and cancer. Future studies are required to clarify the context−dependent functions of FXR in specific immune cell subsets across different tissues and disease settings.

Recent studies have further established a protective role for FXR in inflammatory bowel disease (IBD). In the intestinal immune milieu, FXR activation ameliorates DSS-induced colitis and helps restore intestinal barrier function. This occurs by inhibiting important transcription factors such as Rorc and Batf, which promote ILC3 differentiation, and by increasing REVERBα (Nr1d1), which suppresses IL-17 transcription (30). Furthermore, FXR modulates innate immune sensing pathways. The agonist GW4064 protects against lipopolysaccharide (LPS)-induced ileocolitis by directly inhibiting the TLR4/MyD88 axis, reducing the expression of TLR4 and MyD88, attenuating pro-inflammatory cytokine release and macrophage infiltration, and preserving the integrity of epithelial tight junctions (31). Certain BAs, such as 7-ketoLCA and UDCA, may synergistically antagonize FXR to activate Wnt signaling, promoting intestinal stem cell renewal and barrier repair. However, the precise mechanistic interplay remains to be fully elucidated (20). FXR plays a protective role in IBD by inhibiting the pro-inflammatory response of innate immune cells such as ILC3, inhibiting TLR4-mediated signaling, and aiding in the maintenance and repair of the intestinal epithelial barrier. Future studies should focus on the cell type-specific contributions of FXR to different intestinal compartments during chronic inflammation.

FXR is widely recognized as the central regulator of BA homeostasis. In hepatocytes, FXR activation induces the transcription of the SHP, which suppresses cholesterol 7α-hydroxylase (CYP7A1), the rate-limiting enzyme in BA synthesis (32). In parallel, FXR signaling in the intestine strongly stimulates expression of fibroblast growth factor 15 (FGF15) in rodents and its human counterpart FGF19 (33). Circulating FGF15/19 subsequently activates FGFR4/β-Klotho complexes in hepatocytes, leading to c-Jun N-terminal kinase (JNK) phosphorylation (34). Collectively, these pathways repress the transcription of CYP7A1 and sterol 12α-hydroxylase (CYP8B1), limiting BA production and promoting gallbladder bile storage (35). FXR also plays a crucial role in controlling lipid and glucose balance. Overall, FXR activation lowers circulating lipid levels by inhibiting *de novo* fatty acid synthesis (36), reducing hepatic very-low-density lipoprotein (VLDL) secretion (37), and enhancing triglyceride hydrolysis, lipid clearance, and fatty acid oxidation (38). Besides its effects on lipid metabolism, FXR signaling improves glucose homeostasis by attenuating hepatic gluconeogenesis and glycolysis while promoting glycogen synthesis (39). Mechanistically, the suppression of gluconeogenesis is mediated by the SHP-dependent inhibition of key gluconeogenic transcription factors (40).

Based on the immunomodulatory mechanisms of FXR, therapeutic strategies targeting FXR have shown promise for treating related disorders, including nonalcoholic steatohepatitis (NASH), primary biliary cholangitis (PBC), primary sclerosing cholangitis (PSC), and type 2 diabetes (41–44). Several FXR−targeting agents have advanced to Phase II clinical trials. Obeticholic acid (OCA), already approved for PBC, and other candidates such as cilofexor, EDP−305, nidufexor, tropifexor, and TERN−101 are being evaluated for NASH, PBC, PSC, and liver fibrosis (45). Despite demonstrating efficacy, the clinical translation of these agents is often limited by dose−dependent, on−target adverse effects. Pruritus is a frequently reported side effect, observed with OCA, EDP−305, tropifexor, cilofexor, and TERN−101 (46). Additionally, unfavorable lipid alterations, such as elevated low-density lipoprotein or reduced high-density lipoprotein, have been noted with agents like tropifexor and cilofexor at higher doses, narrowing their therapeutic window (47). These findings underscore a consistent trade-off between efficacy and tolerability in systemic FXR activation. Future strategies should prioritize optimizing dosing regimens and investigating combination therapies to augment therapeutic benefits while minimizing adverse effects. Nevertheless, long-term clinical studies will be imperative to validate their safety and enduring efficacy.

### 2.2 TGR5

TGR5 (also known as GPBAR1) is a G protein-coupled receptor that serves as a key membrane receptor for BAs (48). It is widely expressed in various tissues, with particularly high levels in intestinal epithelium, biliary system, gallbladder, as well as in the placenta and spleen (49). As a primary BA-sensing receptor, TGR5 is activated by several endogenous BAs, with LCA, DCA, CDCA, UDCA, CA, 3-oxoDCA, 3-oxoLCA, alloLCA, isoalloLCA, and 3-oxoUDCA (16, 50). TGR5 activation triggers intracellular cAMP signaling, which regulates a spectrum of physiological processes, including energy metabolism, glucose homeostasis, and gastrointestinal motility (48). Within the innate immune system, TGR5 is abundantly expressed in monocytes, macrophages, and DCs, but is absent in T and B cells (51).

TGR5 activation generally exerts anti-inflammatory effects on the innate immune system (**Figure 2**). In macrophages, TGR5 activated by LCA and DCA suppresses the production of pro-inflammatory cytokines, such as TNF-α, dependent on the cAMP-dependent pathway (52). This anti-inflammatory effect is also observed with 3-oxoDCA, which ameliorates colitis in part through TGR5 by counteracting RORγt agonist or IL-23-driven pathology (50). Multiple BAs, including LCA, DCA, 3-oxoLCA, and isoLCA, act as TGR5 agonists to promote macrophage polarization toward the M2 phenotype (53, 54). Besides macrophages, TGR5 signaling modulates the activity of other innate immune cells. DCA binding to TGR5 drives DCs toward a tolerogenic phenotype and reduces their ability to express costimulatory molecules and secrete pro-inflammatory cytokines (55). In NK cells, isoLCA-mediated TGR5 activation inhibits the downstream cAMP-PKA-CREB1 axis. This leads to reduced CREB1 phosphorylation, impairing NK cell cytotoxicity and thereby promoting immune evasion in hepatocellular carcinoma (HCC) (56). Overall, TGR5 activation by various BAs coordinates a multicellular anti-inflammatory program, suppressing pro-inflammatory responses in macrophages and DCs while exerting context-dependent regulation of cytotoxic immunity in NK cells. Developing cell-selective TGR5 modulators is crucial for translating these immunomodulatory effects into targeted therapies for inflammatory diseases and cancer.

### 2.3 VDR

VDR is a nuclear receptor crucial for maintaining immune homeostasis and has well-characterized anti-inflammatory and antitumor functions (57). It is expressed in a broad range of immune cells, including T cells, B cells, monocytes, and macrophages (58). In the innate immune system, VDR activation is suppressed in various cell types. It decreases neutrophil extracellular trap formation by inhibiting chemokine production, such as CXCL1 and CCL20 from neutrophils (59). Furthermore, it reduces the production of pro-inflammatory cytokines in monocytes and macrophages by targeting MAPK phosphatase-1 (60). VDR also suppresses the IgE receptor FcϵRI and the PI3K/Akt/p38 MAPK/HIF-1α pathway in mast cells, thereby limiting their activation and VEGF release (61). Furthermore, it hinders DC maturation (62). During adaptive immunity, iNKT cell development depends on the VDR (63). By suppressing the production and response of IL-2, VDR also restricts the homeostatic proliferation of naïve CD8^+^ T cells, preventing their abnormal activation and pathogenic conversion, and maintaining intestinal immune homeostasis (64). Moreover, a combination of the LCA and 3-oxoLCA—but not the canonical ligand calcitriol—restored colonic RORγ^+^ Treg cells in a colitis model (65). Supplementation with 12-ketolithocholic acid (12-KLCA) reversed the downregulation of VDR in ILC3s induced by DSS and reduced IL-17A secretion, although the precise mechanism regulating VDR mRNA levels remains unknown (66). Considering the extensive involvement of VDR in inflammation, autoimmunity, and cancer, and its selective activation by secondary bile acids such as LCA and specific oxidized derivatives, targeting VDR represents a promising therapeutic strategy.

### 2.4 RORs

RORs are ligand-dependent transcription factors essential for immune development and homeostasis and are expressed in the thymus, lung, liver, kidney, skeletal muscle, and brown adipose tissue. The ROR family comprises three members: RORα, RORβ, and RORγ, encoded by distinct genetic loci (67). RORγ gives rise to a key splice variant, RORγt, which is selectively expressed in immune cells, such as Th17 cells, Tregs, and ILC3s (68, 69). RORγt is a master transcriptional regulator that drives IL-17A expression and is pivotal for Th17 cell differentiation (70). By modulating the balance between pro-inflammatory Th17 cells and suppressive Tregs, RORγt plays a central role in maintaining intestinal immune homeostasis and orchestrating responses to commensal microbiota (71). Several secondary BA derivatives—including 3-oxoDCA, 3-oxoLCA, alloLCA, and isoalloLCA, exert anti-inflammatory effects by directly inhibiting RORγt-dependent transcriptional activity. This suppression limits Th17 cell differentiation and, in concert with TGR5 activation, promotes a coordinated anti-inflammatory immune response (72).

### 2.5 Other receptors

Besides the established canonical signaling pathways, several non-canonical BA receptors have been identified. Initially classified as an orphan nuclear receptor, NR4A1 modulates the immune system by regulating T cell differentiation and survival (73). Microbial BA derivatives, such as isoalloLCA, trigger mitochondrial reactive oxygen species (ROS) production and NR4A1-dependent chromatin remodeling at the FoxP3 locus, promoting the differentiation of naïve CD4^+^ T cells into Tregs, which exert anti-inflammatory effects (74). CAR is a nuclear receptor that functions as a xenobiotic sensor in the liver and intestine. CAR plays a role in the glycolithocholic acid (GLCA)-induced expansion of Tregs (75). A recent study identified a subset of lithocholic acid derivatives, such as 3-oxoLCA and 3-oxo-Δ^4,6^-LCA, as potent hAR antagonists. Furthermore, 3-oxo-Δ^4,6^-LCA enhances the recruitment of tumor-infiltrating CD8^+^ T cells exhibiting stem-like properties in an androgen-dependent manner (76).

Besides the receptors discussed above, for which direct or indirect evidence supports their role in BA-mediated immune regulation, several other BA receptors have also been shown to influence immune function in response to synthetic chemicals or alternative ligands (**Figure 2**). The steroid and xenobiotic receptor (SXR) in humans, along with its rodent equivalent, pregnane X receptor (PXR), pairs with RXR and uses BA as a ligand (77). PXR has been activated *in vitro* by multiple BA species, including LCA, DCA, 3-oxoLCA, GDCA, acetylated derivatives of CA and DCA, and glutamate-conjugated CA and CDCA (78–80). PXR functions as a transcriptional regulator of NLRP3. Activation of PXR by the agonists rifampicin and SR12813 enhances NLRP3 expression in vascular endothelial cells, subsequently promoting caspase-1 cleavage and IL-1β maturation, consistent with a pro-inflammatory response (81). PXR activation is further associated with increased expression of multiple pattern recognition receptor genes, including TLR2, TLR4, TLR9, NOD1, NOD2, and NLRP1, underscoring the prominent role of PXR in the regulation of innate immune responses (81). In adaptive immunity, PXR functions predominantly as a negative regulator by suppressing T lymphocyte activation and proliferation, limiting interferon-γ (IFN-γ) production, and restraining B-1 cell development (82, 83). Liver X receptor (LXR) is another nuclear receptor composed of two RXR-dependent isoforms with distinct tissue expression patterns, including ubiquitously expressed LXRβ and tissue-enriched LXRα, and responds to activation by hyodeoxycholic acid (HDCA) and its derivatives (84, 85). Activation of LXR by alternative agonists exerts immunological effects, most notably anti-inflammatory actions in monocytes and macrophages through innate immune pathways (86, 87); beyond innate immunity, LXR also contributes to the regulation of multiple T cell subsets (88). Collectively, these findings highlight PXR and LXR as emerging regulators of immune function beyond classical BA receptors. Future studies should further delineate the distribution and cell type-specific roles of these receptors across diverse immune cell populations, identify additional BA species capable of modulating their activity, and clarify how PXR- and LXR-dependent signaling ultimately integrates into BA-mediated regulation of the immune system.

## 3 Microbial transformation: the bridge connecting gut microbiota and immune regulation

Microbial transformation is the primary source of chemical diversity in the BA pool (89). Initially, conjugated BAs from the liver are deconjugated by microbial bile salt hydrolases (BSHs) to release free BAs. These free BAs then serve as substrates for microbial 7α-dehydroxylation, converting primary BAs into secondary BAs, a key step that markedly increases hydrophobicity. Further structural diversification occurs through oxidation and epimerization reactions catalyzed by hydroxysteroid dehydrogenases (HSDHs), which modify the stereochemistry of hydroxyl groups to generate bioactive derivatives. Finally, certain bacteria can reconjugate BAs with non-canonical amino acids to produce a range of microbially conjugated BAs (90). This cascade of interconnected microbial modifications fundamentally reshapes the composition and physicochemical properties of the host BA pool. The resulting diverse BA metabolites acquire critical signaling functions that regulate essential physiological processes, including BA homeostasis, intestinal barrier integrity, immune response, and energy metabolism.

### 3.1 Gut microbial regulation of BA metabolism

Conjugated primary BAs undergo an initial and essential microbial-mediated deconjugation step after their secretion into the intestinal lumen. This hydrolysis, catalyzed by BSHs from genera such as *Lactobacillus*, *Bifidobacterium*, *Bacteroides*, and *Clostridium*, releases free BAs along with glycine or taurine (91). The resulting free BAs are further diversified by position-specific HSDHs, including 3α-, 7α/7β-, and 12α/12β-HSDHs, produced by various gut bacteria. Structurally, HSDHs belong to three major protein superfamilies: short-chain dehydrogenases/reductases, medium-chain dehydrogenases/reductases, and aldo-keto reductases (92). These enzymes catalyze reversible oxidation, reduction, and epimerization reactions that interconvert α- and β-hydroxyl groups at the C3, C7, and C12 positions (93). In a more specialized pathway, the BA-inducible operon, primarily found in *Clostridium scindens* and *C. hiranonis*, mediates 7α−dehydroxylation of CA and CDCA to generate the classical secondary BAs such as DCA and LCA (11, 94).

### 3.2 BA-mediated regulation of the gut microbiota

BAs are key regulators of the gut microbial ecosystem (95). In the proximal small intestine, BAs form mixed micelles that help control bacterial density (96). Their antimicrobial activity is selective: while gram-negative and some resistant species are largely protected from BA-induced membrane disruption, DNA damage, and cell death due to enhanced intrinsic defenses (97). More susceptible taxa, such as *Spirochetes*, *Staphylococcus*, *Streptococcus pneumoniae*, and *Enterococcus*, experience dose-dependent membrane injury. Unconjugated BAs possess stronger antimicrobial potency than conjugated BAs (98, 99). Conversely, BAs deficiency in the gut can lead to bacterial overgrowth and increased pathogen colonization (100).

The modulation of BA levels directly impacts microbiota composition. Inhibition of BA synthesis by OCA promotes the expansion of gram-positive bacteria in mice, including *Lactobacillus*, *Bifidobacterium*, and *Lactococcus*. In contrast, CA supplementation reduces the abundance of *Lactobacillus* and *Ruminococcus*, while favoring gram-negative pathobionts such as *Prevotella* and *Desulfovibrio* (101). Similarly, in healthy humans, UDCA supplementation increases the abundance of *Faecalibacterium prausnitzii* and decreases that of *Ruminococcus gnavus* (102). With the recent discovery of numerous novel BAs, their functional roles, particularly their specific effects on gut microbiota, have become the focus of growing research interest.

### 3.3 Microbial enzymatic pathways linking BA metabolism to immune regulation

Emerging studies are bridging this gap through targeted genetic and enzymatic research. Deletion of BSH genes—but not 7α-HSDH genes—in *Bacteroides thetaiotaomicron* and *B. fragilis* reduced their capacity to induce colonic RORγ^+^ Tregs, indicating that microbiota-produced free primary BAs act as key signaling molecules in regulating the RORγ^+^Treg compartment (65). Paik et al. demonstrated that bacterial 3α-HSDH (from species such as *Ruminococcus gnavus* and *Eggerthella lenta*) converts LCA to 3-oxoLCA, which is then transformed by 3β-HSDH into isoLCA. Both metabolites are potent RORγt antagonists that suppress Th17 cell differentiation (8, 72). Similarly, *Parabacteroides merdae* and *Bacteroides dorei* can convert LCA to isoalloLCA through a sequential enzymatic cascade involving a 5β-reductase (producing 3-oxo-Δ^4^-LCA), a 5α-reductase (yielding 3-oxoalloLCA), and finally a 3β-HSDH, which promotes the differentiation of Treg cells (74).

Besides classical pathways, microbial modifications create entirely novel immunomodulatory BA species. A key example is sulfation, a detoxification route that can be exploited by commensals to produce sulfated cholic acid (CA7S). Notably, CA7S was recently identified as an endogenous agonist of mucosal-associated invariant T (MAIT) cells, revealing a novel microbiota-dependent BA-immune axis (103–106). However, the complexity and interdependence between the gut microbiota and BA pool means that few studies have systematically linked a specific microbial strain to a defined BA metabolite and, ultimately, to a precise immune outcome. Most studies describe broad shifts in microbial communities and BA profiles without establishing direct causal strain-metabolite-immunity relationships. Future research should prioritize the establishment of causal mechanistic chains to fully exploit the therapeutic potential of the gut microbiota-BA-immune network, from bacterial genes to BA metabolites, immune receptors, and cell fate.

Current therapeutic strategies targeting BAs through microbial modulation broadly fall into two categories: using engineered bacteria to produce specific BAs and administering exogenous probiotics to reshape the gut microbiota for beneficial BA metabolism. The direct approach involves the use of engineered bacteria. Codon-optimized genes encoding 3α-HSDH and 3β-HSDH were integrated into *Bacteroides thetaiotaomicron*, allowing the conversion of DCA to isoDCA, which promotes colonic pTreg cell differentiation and maintains mucosal immune tolerance (19). An alternative strategy involves the administration of specific probiotics. Administering *Bacteroides uniformis* in a DSS-induced colitis mouse model shifted the microbiota composition by promoting beneficial genera, such as *Bacteroides* and *Bifidobacterium*, and decreasing potential pathogens. This shift was associated with elevated levels of specific BAs that inhibit Th17 cell differentiation, including α-MCA, HDCA, and isoLCA (107). Similarly, *Bifidobacterium breve* has demonstrated antitumor potential, partly by deconjugating TCA to CA via its BSH activity while favorably modulating microbial composition (108).

Broader microbial interventions, such as fecal microbiota transplantation (FMT), also exert effects through the BA axis. FMT from healthy to ovariectomized rats restored microbial diversity and increased intestinal level of GLCA, which correlated with an increased frequency of circulating Tregs and ameliorated osteoporosis symptoms (75). Dietary interventions may have similar effects. Exclusive enteral nutrition increased the abundance of BA-metabolizing bacteria (e.g., *Clostridium innocuum*, *Hungatella hathewayi*) and elevated specific BA levels, including hyocholic acid (HCA). HCA inhibited TNF-α production by CD4^+^ T cells from patients with Crohn’s disease, suggesting a direct immunomodulatory link (109). These studies exemplify the translational potential of targeting the microbiota-BA axis, moving from correlation to causation by linking specific microbial functions to defined BA metabolites and immune outcomes. Future development of next-generation probiotics, engineered symbionts, and precise dietary formulas will depend on a deeper mechanistic understanding of these tripartite interactions to design effective, context-specific therapies for immune and metabolic disorders.

## 4 Dual immunological roles of BAs

Recent studies have identified distinct BA subsets that preferentially promote immune tolerance and regulatory pathways, or, conversely, drive pro-inflammatory immune activation. In addition, a subset of BAs displays dual, context-dependent immunomodulatory effects on specific immune cell populations, including CD4^+^ T cells, CD8^+^ T cells, macrophages, and NK cells (**Figure 1**). Here, we summarize the central role of BAs within the immunometabolism network (**Table 1**), which provides new insights for targeting BA signaling pathways in the treatment of immune-related diseases.

**Table 1:** Classification of the immunomodulatory functions of bile acids.

Classification	Bile acids	Effect on immune cell	Key mechanism	Disease or model
Regulatory	isoDCA	Promotes colonic Treg differentiation (19)	Antagonizes FXR of DCs, promotes RORγt^+^ Treg cells in a CNS1-dependent manner	C57BL/6 mice
	DCA, LCA	Suppress macrophage and the production of pro-inflammatory factors such as TNF-α (52)	Activate the TGR5-cAMP pathway in macrophages	Crohn’s disease
	alloLCA, isoalloLCA, 3-oxoDCA, 3-oxoLCA	Promote M2 macrophage polarization, promote Treg differentiation and inhibit Th17 differentiation (50)	Activate TGR5 and antagonize RORγt	IBD
	3-oxoLCA	Inhibits Th17 differentiation (8)	Directly binds to RORγt and suppresses its transcriptional activity	IBD
	isoalloLCA	Promotes Treg differentiation (8)	Increases mitoROS thus enhances H3K27 acetylation at FoxP3 promoter via CNS3 enhancer	IBD
	alloLCA	Suppresses M1 and promotes M2 phenotype of macrophages, inhibits Th17 differentiation and promotes Treg differentiation (53)	Activates TGR5 and antagonizes RORγt	MASH
	DCA, LCA	Promote Treg differentiation and inhibit Th17 cells, shift macrophages from M1 to M2 phenotype (55)	DCA binds GPBAR1 on DCs, LCA acts as RORγt inverse agonists	RRMS
	3-oxoLCA, isoLCA	Inhibit Th17 differentiation (72)	Directly bind to RORγt	IBD
	isoalloLCA	Enhances Treg differentiation (74)	Acts on NR4A1 binding to Foxp3 promoter, increases Foxp3 transcription.	IBD
	α-MCA, HDCA, isoLCA	Inhibit Th17 cell differentiation (107)	Significantly reduce the mRNA levels of TNF-α, IL-6 and IL-17A	IBD
	NorUDCA	Inhibit Th17 cell differentiation and enhances Treg differentiation (111)	Inhibit mTORC1 signaling in T cells, reprogram T cell metabolism	Primary sclerotic cholangitis
	GUDCA	GUDCA-treated keyhole limpet hemocyanin mice showed expanded CCR7^-^PD-1^+^ memory Tfh cells (112)	unknown	Neuromyelitis Optica Spectrum Disorder (NMOSD)
	CA, CDCA, UDCA	Specific mixtures primary BAs restore the pool of colonic Foxp3^+^ Tregs (65)	unknown	IBD
	3-oxo-Δ^4,6^-LCA	Promotes antitumor immunity by enhancing stem-like CD8+ T cells (76)	Promotes anti-PD-1 immunity therapy in an androgen-dependent manner	Bladder carcinoma
	TLCA	Enhances the antitumor effect of CD8^+^ T cells (115)	Increases proliferation and upregulates cytokines and signaling molecules (JAK1, STAT1)	Non-small cell lung cancer
	TCA	Promotes M2 macrophage polarization (17)	Activates FXR in macrophages	HCC
	T-β-MCA, CDCA	Promote NKT cell activation and IFN-γ production (126)	Increase CXCL16 expression in liver sinusoidal endothelial cells, enhance CXCR6^+^ NKT cell recruitment to the liver	HCC
	12-ketoLCA	Influences ILC3 (66)	Suppresses IL-17A secretion from colonic ILC3s by upregulating VDR expression.	IBD
Inflammatory	GCDCA	Inhibits T cell proliferation (115)	Downregulates activation markers (IFN-γ, TNF-α, GZMB)	Non-small cell lung cancer
	DCA	Suppresses antitumor CD8^+^ T cell effector functions (116)	Inhibits the Ca^2+^-NFAT2 signaling pathway by enhancing plasma membrane Ca²^+^-ATPase activity	CRC
	DCA	Promotes M1 macrophage polarization (122)	Binds to M2 muscarinic acetylcholine receptor, activated Src kinase, upregulates TLR2 transcription via AP-1, TLR2 activation leads to tyrosine phosphorylation and downstream, NF-κB/ERK/JNK signaling	High-fat diet -induced colonic inflammation
	TCDCA, GCDCA	Inhibit CD8^+^ T cell cytotoxicity (117)	Induce oxidative stress, reduce mitochondrial respiration, increase ROS in CD8^+^ T cells, leading to T cell death.	HCC
	LCA, isoalloLCA	Promote T cell dysfunction (117)	Activate ER stress, upregulate NR4A1/2, inhibit cytokine production (IFN-γ, TNF-α)	HCC
	TCA	Inhibits CD8^+^ T cell infiltration and function (108)	Blocks ERK phosphorylation via the MAPK pathway, reduces expression of cytotoxic cytokines	HCC
	TCA	Impairs effector functions of CD3^+^CD8^+^ T and NK cells (118)	unknown	Chronic hepatitis B
	CDCA	Inhibits M2 macrophage polarization (121)	unknown	AML
	ω-MCA GLCA	Reduce NKT cell recruitment to the liver (126)	Decrease CXCL16 expression in liver sinusoidal endothelial cells	HCC
	isoLCA	Impairs NK cell cytotoxicity (56)	isoLCA was dependent on phosphorylated CREB	HCC
	GCDCA	Skews monocytes toward a pro-inflammatory phenotype (127)	Increases pro-inflammatory markers (CD16, CD80) and promotes a tumor associated macrophage, likely involvesTGR5 and FXR.	Primary sclerotic cholangitis
	CA, CDCA	Induced eosinophil accumulation (27)	CA and CDCA-induced ILC2 activation promoted IL-33 expression, acted via FXR receptor	IBD
	T-β-MCA, GCA	Inhibit T cell function (128)	Promote Arg1 and iNOS expression inneutrophils via the p38 MAPK pathway	CRC liver metastasis

### 4.1 CD4^+^ T cells

**Figure 3:**
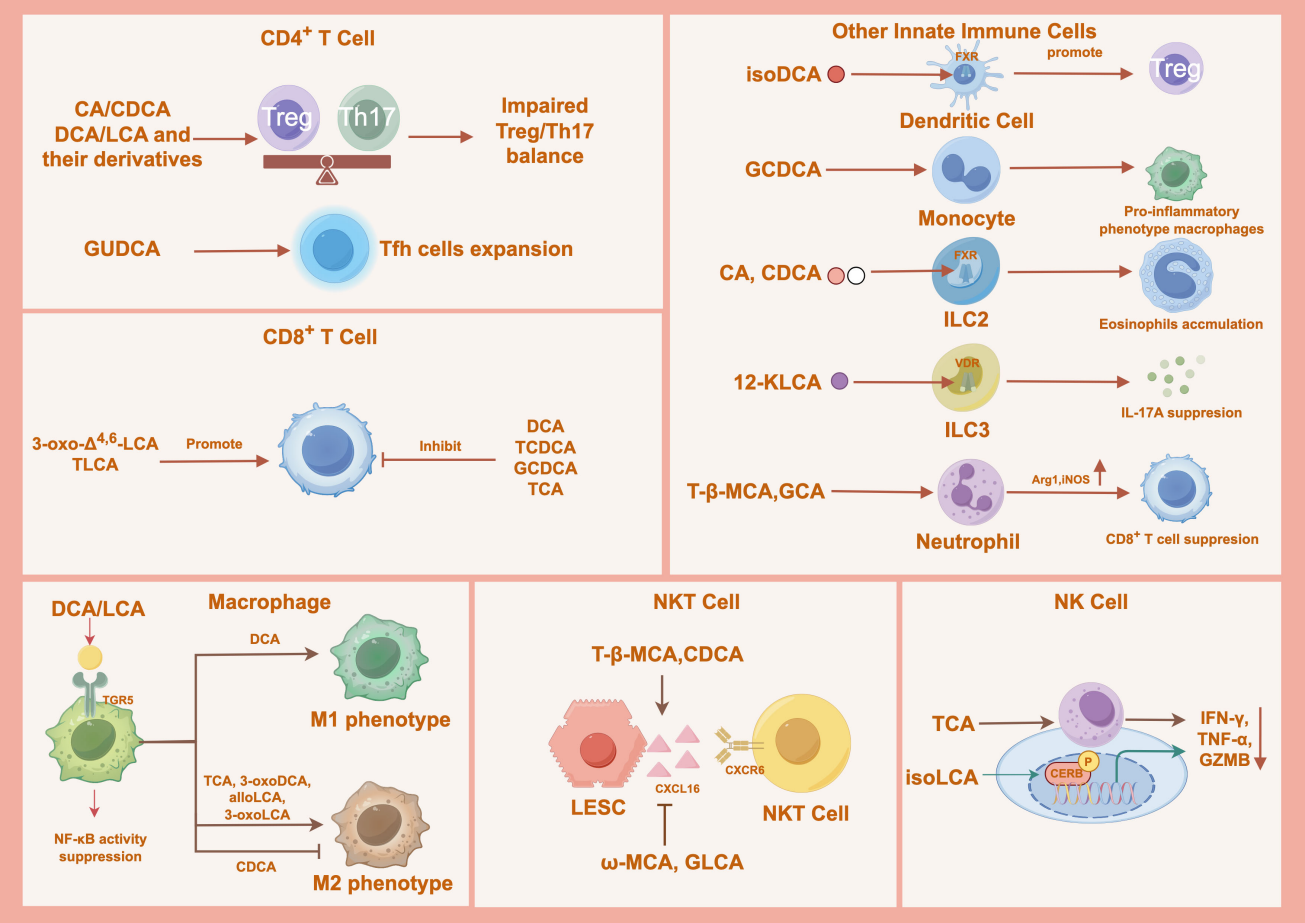
Cell type-specific immunomodulatory effects of bile acids across innate and adaptive immune cells. Bile acids differentially regulate adaptive and innate immune cell subsets, including CD4^+^ and CD8^+^ T cells, dendritic cells, macrophages, innate lymphoid cells, neutrophils, and NK/NKT cells. Through bile acid-specific signaling, these pathways modulate T cell differentiation, cytotoxic function, innate immune activation, and immune cell recruitment, collectively shaping immune homeostasis and inflammatory responses in a context-dependent manner. CA, Cholic Acid; CDCA, Chenodeoxycholic Acid; LCA, Lithocholic Acid; DCA, Deoxycholic Acid; GUDCA, Glycoursodeoxycholic Acid; 3-oxo-Δ^4,6^-LCA, 3-oxo-Δ^4,6^-lithocholic Acid; TLCA, Taurolithocholic Acid; TCDCA, Taurochenodeoxycholic Acid; GCDCA, Glycochenodeoxycholic Acid; TCA, Taurocholic Acid; 3-oxoDCA, 3-oxodeoxycholic Acid; alloLCA, allo−lithocholic Acid; 3-oxoLCA, 3-oxolithocholic Acid; isoDCA, Isodeoxycholic Acid; 12-KLCA, 12-ketolithocholic acids; GCA, Glycocholic Acid; T-β-MCA, Tauro-β-muricholic Acid; isoLCA, Isolithocholic Acid; GLCA, Glycolithocholic Acid; ω-MCA, ω-muricholic Acid; NKT, natural killer T; Th17, T helper 17 cells; Tregs, regulatory T cells; Tfh, T Follicular Helper; GZMB, Granzyme B; LESC, Liver Sinusoidal Endothelial Cells; TNF-α, Tumor Necrosis Factor α; IFN-γ, Interferon-γ.

CD4^+^ T lymphocytes, or helper T cells, are central orchestrators of adaptive immunity. Their remarkable plasticity allows them to differentiate into distinct effector subsets—including Th1, Th2, Th17, Treg, Th9, Th22, and T follicular helper (Tfh) cells, each defined by a unique cytokine profile that directs immune responses toward defense, tolerance, and homeostasis (110). Secondary BAs, such as DCA, LCA, and their derivatives (e.g., 3-oxoDCA, 3-oxoLCA, alloLCA, isoalloLCA, isoLCA, and 3-oxoUDCA), promote Treg differentiation while suppressing Th17 cell development (**Figure 3**) (8, 50, 53, 55, 72, 74). Conversely, α-MCA, HDCA, and isoLCA directly inhibit Th17 cell differentiation (107). NorUDCA further shifts this balance by driving the trans differentiation of Th17 cells into Tregs through inhibition of the glutamine-mTORC1-glycolysis axis (111). Primary BAs also contribute: CA and CDCA foster Treg differentiation, whereas LCA combined with 3-oxoLCA helps restore colonic RORγ^+^ Tregs (50, 65). Besides the Th17/Treg axis, BAs modulate other T-cell subsets. Glycoursodeoxycholic acid (GUDCA) upregulates the chemokine CXCL13, promoting the homing and differentiation of CXCR5^+^ Tfh precursor cells in germinal centers of secondary lymphoid organs (**Figure 3**). This mechanism enhances Tfh expansion and has been linked to relapse of neuromyelitis optica spectrum disorder (112).

### 4.2 CD8^+^ T cells

CD8^+^ T cells play a critical role in immune surveillance by recognizing and eliminating virus-infected cells, intracellular bacteria, and malignant cells (113). Their cytotoxic function is executed through perforin/granzyme-mediated cell death and death receptor pathways, such as Fas-FasL, and is amplified by the secretion of pro-inflammatory cytokines, such as IFN−γ and TNF−α (114). In certain cancer types, BAs can bolster antitumor immunity (**Figure 3**). 3-oxo-Δ^4,6^-LCA enhances stem-like properties in CD8^+^ T cells and synergizes with PD−1 blockade via AR signaling (76). In non−small cell lung cancer (NSCLC), taurolithocholic acid (TLCA) promotes CD8^+^ T cell proliferation, cytokine production, and memory differentiation, whereas glycochenodeoxycholic acid (GCDCA) exhibits immunosuppressive effects (115).

Conversely, several BAs suppress CD8^+^ T cell responses and promote immune evasion (**Figure 3**). DCA attenuates CD8^+^ T cell activation by disrupting Ca²^+^-NFAT2 signaling (116). In HCC, taurochenodeoxycholic acid (TCDCA) and secondary BAs, such as LCA, exacerbate CD8^+^ T cell exhaustion through oxidative stress and endoplasmic reticulum stress (117). TCA functions as a shared immunosuppressive metabolite in chronic hepatitis B and obesity-associated HCC, impairing CD8^+^ T cell effector function and survival. Microbial deconjugation of TCA by *Bifidobacterium breve* can restore CD8^+^ T cell activity, underscoring the therapeutic potential of modulating BA metabolism (108, 118). Thus, BAs act as context-dependent regulators of CD8^+^ T cell immunity, with select metabolites enhancing antitumor responses, whereas others drive exhaustion. Future studies should focus on developing strategies to selectively inhibit exhaustogenic BAs or administer immunostimulatory BA species to improve cytotoxic T cell function in infections and cancer.

### 4.3 Macrophages

Macrophages are highly plastic innate immune cells that are central to tissue homeostasis, host defense, and inflammation regulation (119). They polarize into distinct functional states: classically activated (M1) macrophages exhibit pro−inflammatory properties and secrete cytokines, such as TNF−α, IL−6, and IL−12; alternatively activated (M2) macrophages promote tissue repair and resolution of inflammation through the production of IL−10 and TGF−β (120). BAs critically influence macrophage polarization and function, often exerting anti−inflammatory effects (**Figure 3**). In Crohn’s disease, DCA and LCA activate TGR5 signaling in macrophages, which suppresses NF−κB activity and reduces the production of pro−inflammatory cytokines, such as TNF−α (52). Similarly, secondary BAs and derivatives, including 3−oxo−DCA, alloLCA, and 3−oxoLCA, promote a shift toward the M2 phenotype both *in vitro* and *in vivo*, characterized by downregulation of M1 markers (e.g., CD38 and TNF−α) and upregulation of the M2 marker CD206, attenuating colonic inflammation and supporting epithelial barrier integrity (50). In HCC, elevated TCA levels driven by Sirt5 deficiency enhance M2 polarization, contributing to an immunosuppressive tumor microenvironment (17). AlloLCA also effectively inhibits M1 polarization promotes an anti−inflammatory M2 phenotype, and alleviates hepatic inflammation, steatosis, and fibrosis (53).

Conversely, certain BAs exert pro−inflammatory effects on macrophages (**Figure 3**). CDCA interferes with IL−4−induced M2 polarization, impairing the macrophage−mediated support of leukemia cell proliferation (121). Moreover, DCA transactivates TLR2 via the M2 muscarinic acetylcholine receptor and Src signaling, while also upregulating TLR2 transcription through AP−1. TLR2 activation drives M1 polarization via downstream NF−κB, ERK, and JNK pathways, exacerbating colonic inflammatory injury (122). Therefore, BAs function as context-dependent switches for macrophage polarization and can either resolve or exacerbate inflammation.

### 4.4 NK and NKT cells

NK cells are critical innate immune effectors that secrete cytokines, such as IFN-γ and TNF-α, and mediate cytotoxicity through perforin and granzyme B (GZMB). Within tumors, NK cell infiltration is often reduced, and this deficiency is associated with disease progression and poor survival (123). Certain BAs suppress NK cell activity. TCA impairs cytotoxicity by reducing GZMB and perforin production (118). IsoLCA also attenuates NK cell function in a pCREB-dependent manner (56). NKT cells represent a distinct subset of innate-like lymphocytes that express features of both NK cells and conventional T cells, thereby bridging innate and adaptive immunity and playing an important role in tumor immune responses (124, 125). CDCA and tauro-β-muricholic acid (T-β-MCA), exert antitumor effects by upregulating chemokine CXCL6, which promotes the hepatic recruitment of CXCR6^+^ NKT cells and suppresses liver tumor growth. In contrast, ω-MCA and GLCA negatively correlate with CXCL16 levels, suggesting a limitation on NKT cell recruitment that may favor tumor progression (126). Collectively, BAs act as context-dependent regulators of NK and NKT cell biology by differentially influencing their cytotoxic functions and tumor recruitment (**Figure 3**). Future studies should elucidate how tissue-specific BA compositions shape NK and NKT cell phenotypes across distinct tumor microenvironments and evaluate combinatorial strategies that counteract immunosuppressive BAs while harnessing immunostimulatory species to potentiate NK/NKT-mediated antitumor immunity.

### 4.5 Other innate immune cells

BAs also modulate the function of other innate immune cells, including monocytes, ILCs, and neutrophils, influencing the inflammatory process in related diseases (**Figure 3**). When co-administered with human recombinant M-CSF, GCDCA increases the proportion of cells expressing CD16^+^, CD80^+^, and CD163^+^ markers. In LPS-stimulated monocytes, it further expands the CD163^+^ population, suggesting a role in skewing monocytes toward a pro-inflammatory phenotype (127). In neutrophils, primary BAs upregulate Arg1 and iNOS expression via the p38 MAPK pathway, such as T-β-MCA and GCA. This alteration suppresses T cell activity and promotes liver metastasis in colorectal cancer (128).

## 5 Tissue-specific immunomodulation: from local barriers to systemic diseases

As a systemic metabolic signaling molecule, it elicits tissue-specific immune responses across multiple organ systems, contributing to the regulation of homeostasis and disease in the gut, liver, nervous system, and various immune microenvironments (**Figure 1**). These differential effects arise from region-specific variations in BA composition, receptor distribution, immune cell metabolic states, and local microbiota. Systematic mapping of these context-dependent interactions is essential for understanding how BA signaling integrates immune, metabolic, and microbial networks in health and disease. The following sections detail the specific mechanisms by which BAs modulate immune functions within each key system.

### 5.1 Intestine: a critical interface for immune homeostasis

The intestine, where BA concentrations peak and microbial interactions are most active, is the primary site of BA−mediated immune regulation. Elevated CA level suppresses the proliferation of Lgr5^+^ intestinal stem cells, potentially aggravating epithelial injury (129). Primary BAs, such as TCA, GCA, and GCDCA, are markedly increased, whereas secondary BAs, such as LCA and DCA are significantly reduced, reflecting the depletion of microbial taxa capable of secondary BA transformation in IBD patients (130). In UC, a similar shift toward primary BAs and away from secondary BAs further disrupts epithelial tight junctions, perpetuating the cycle of inflammation and barrier dysfunction (131).

Therapeutically, alterations in the BA pool are closely linked to treatment outcomes in IBD patients. Patients who respond to anti−TNF therapy exhibit higher circulating levels of secondary BAs, whereas non−responders exhibit elevated levels of unconjugated primary BAs (132). Preclinical studies demonstrated that norUDCA, UDCA, and TUDCA attenuate colitis in murine models (22, 111, 133). Notably, the FXR agonist OCA alleviates intestinal inflammation and enhances barrier integrity (134). Although LCA can protect against colitis, its clinical utility is limited by associated weight loss, likely due to BA−induced increases in energy expenditure (135). These findings highlight the need to balance the metabolic and immunomodulatory effects of BAs when designing therapies for IBD.

### 5.2 Liver: a metabolic and immune hub

As the primary site of BA synthesis and enterohepatic recirculation, the liver is exposed to high concentrations of BAs that exert direct and potent immunomodulatory effects. BAs function as central rheostats in liver immunity and serve as pathogenic mediators, diagnostic biomarkers, and therapeutic targets. Clinically, impaired bile flow leads to BA retention and cholestatic injury (136). Within hepatocytes, BA accumulation primarily drives pathology by inducing endoplasmic reticulum stress and mitochondrial damage, activating signaling pathways such as TLR9, NFAT, and Egr1 to trigger a pro-inflammatory response rather than direct cytotoxicity (137). BAs also activate hepatic stellate cells to promote liver fibrosis (138).

BA signatures are emerging as valuable diagnostic and prognostic biomarkers for liver diseases. In non-alcoholic fatty liver disease (NAFLD), total BAs and multiple species are significantly elevated. Specific correlations exist, such as TCA with severe steatosis, GCA with inflammation, and total BA/GCA/TCA with fibrosis (139, 140). Elevated serum total BAs are an independent risk factor for HCC (141). Altered BA ratios also show strong predictive value; for instance, increased TCDCA/GCDCA and TDCA/GDCA ratios are associated with a higher future HCC risk in cirrhotic patients, whereas a decreased TCA/CDCA ratio correlates with a lower risk (142). Elevated levels of primary BAs, such as GCA and GCDCA, are associated with an increased HCC risk (142). However, most studies on BAs as biomarkers of liver diseases are in the clinical phenomenon stage, and more in-depth exploration of molecular mechanisms is warranted.

Therapeutic strategies targeting the BA axis focus on the modulation of its synthesis, signaling, and detoxification. UDCA and its derivatives alleviate cholestasis and inflammation (143). FXR agonists, such as OCA, inhibit BA synthesis and stellate cell activation via the FXR-SHP pathway (144), while TGR5 agonists exert anti-fibrotic effects via immunomodulation (145). Dual receptor agonists and FGF19 analogs have shown promise in experimental fibrosis models (146). Furthermore, PPAR modulators, such as elafibranor (a dual PPARα/δ agonist) and seladelpar (a selective PPARδ agonist), reduce BA synthesis and enhance detoxification, offering additional therapeutic avenues (147, 148). Future efforts should focus on refining personalized BA profiling for disease stratification and developing next-generation receptor- or pathway-specific modulators that can precisely rebalance the hepatic immune-metabolic microenvironment without causing systemic toxicity.

### 5.3 Central nervous system: remote immunomodulation via the gut-brain axis

Growing evidence indicates that BAs can cross the blood-brain barrier, a normally restrictive interface that limits the entry of peripheral signals into the CNS (149). Once inside the CNS, BAs engage neural receptors and signaling pathways to modulate neuroinflammation, neurotransmitter homeostasis, and neuronal energy metabolism (150). Their role in neurological disorders is complex and often context-dependent, with shifts in BA composition and signaling having profound effects on disease progression.

In neurodegenerative diseases, such as Alzheimer’s and Parkinson’s, an imbalance characterized by elevated levels of secondary BAs (e.g., DCA and LCA) and reduced levels of neuroprotective BAs (e.g., TUDCA and UDCA) exacerbates neuroinflammation. This imbalance promotes microglial activation, mitochondrial dysfunction, and neuronal apoptosis via receptors such as FXR and TGR5, thereby contributing directly to amyloid-beta deposition, tau pathology, and dopaminergic (151–153). In contrast, neuroprotective effects have been observed under specific conditions. In multiple sclerosis, supplementation with TUDCA or UDCA strongly activates TGR5, inhibiting microglial and infiltrating immune cell overactivation to alleviate demyelination and neuroinflammation (154). DCA and LCA can also reduce CNS infiltration of autoreactive T cells to mitigate neuroinflammation (55). In amyotrophic lateral sclerosis and Huntington’s disease, compensatory elevations of protective BAs, such as UDCA and TUDCA, are often observed in early stages (155, 156). The role of BAs can be dualistic, even within a single disorder. In hepatic encephalopathy, TCA promotes neuroinflammation via the S1PR2 receptor, whereas TGR5 activation may confer protection, highlighting the complexity of BA signaling networks (157, 158). In neuromyelitis optica spectrum disorder, serum levels of GUDCA inversely correlate with relapse rates and with the Tfh-cell activity marker CXCL13 (112). Thus, BAs serve as versatile modulators of CNS immunity and can drive both neurotoxicity and neuroprotection.

The recognition of BAs as pivotal regulators of neuroinflammation and CNS homeostasis has provided promising opportunities for therapeutic innovation and clinical translation in neurology and immunology. Pharmacological agents targeting BA pathways, such as TUDCA, UDCA, and OCA, have demonstrated substantial neuroprotective, anti−inflammatory, and anti−apoptotic effects in diverse models of neurodegenerative diseases. TUDCA and UDCA show therapeutic promise for Alzheimer’s disease, Parkinson’s disease, and multiple sclerosis, largely through their ability to dampen microglial activation and mitigate oxidative stress (159–162). Efforts should be made to elucidate the spatiotemporal dynamics of BA signaling within the brain and develop CNS−penetrant modulators of BA receptors to fully exploit their therapeutic potential in neurological disorders.

### 5.4 Tumor microenvironment: a metabolic tool for immune editing

BAs function as versatile, context-dependent mediators of tumor immunity and can either suppress or enhance antitumor responses based on the specific cancer and microenvironment. The underlying mechanisms exhibit marked organ- and tumor-type specificity, underscoring the diverse and context-dependent roles of BAs in oncology.

In CRC, elevated fecal levels of secondary BAs, particularly DCA, are associated with an increased risk and poorer prognosis (163). Dietary shifts associated with urbanization can alter the microbiome, leading to higher fecal DCA levels that are correlated with CRC prevalence (164). Mechanistically, DCA binds to and activates PMCA4, enhancing calcium efflux in CD8^+^ T cells, suppressing their effector function and promoting tumor growth (116). During CRC liver metastasis, T-β-MCA and GCA upregulate Arg1 and iNOS expression in neutrophils via p38 MAPK signaling, which in turn inhibits T cell activity and supports metastatic colonization (128). In pancreatic ductal adenocarcinoma (PDAC), BAs contribute to pathogenesis through several mechanisms. They regulate the expression of pro-tumorigenic genes such as MUC4 and COX-2, drive mitochondrial dysfunction and NF-κB activation, and promote chronic inflammation that links pancreatitis to PDAC initiation (165, 166). Furthermore, by disrupting calcium homeostasis and acting as iron chelators, BAs induce pancreatic cell necrosis and ferroptosis, exacerbating tissue injury and carcinogenesis (167, 168). As previously discussed, HCC is profoundly influenced by BA dysregulation. In cholangiocarcinoma, TCDCA acts as a TGR5 agonist in cancer-associated fibroblasts and induces CXCL10 secretion. This chemokine promotes tumor progression both directly, by activating the PI3K-AKT pathway in cancer cells, and indirectly, by recruiting and dedifferentiating neutrophils to create an immunosuppressive niche (169). In addition, an altered TCA/TMCA ratio can activate the FXR-FGF15-FGFR4-ERK axis in cholangiocytes, driving their hyperproliferation and contributing to cholangiocarcinogenesis (170). Within the NSCLC tumor microenvironment, TLCA showed synergistic effects with anti-PD-1 therapy, highlighting the potential of BA-mediated immunomodulation to improve immunotherapy outcomes in specific cancer types (115).

BAs possess distinctive structural features, including a rigid, amphiphilic steroidal backbone, that rendering them attractive for biomedical applications. These properties have been increasingly exploited in oncology, particularly for designing biodegradable nanocarriers that enable targeted drug delivery (171). Several BAs and their derivatives have shown therapeutic potential. UDCA enhances the efficacy of anti−PD−1/PD−L1 immunotherapy in HCC and exhibits synergistic anticancer activity when combined with sorafenib (172, 173). OCA, an FXR agonist, delays the progression of metabolic dysfunction−associated steatohepatitis (MASH)−related HCC by modulating the SOCS3/JAK2/STAT3 pathway (174, 175). Preclinical studies further highlight promising antitumor activity and targeted delivery potential for BA derivatives such as BANB−6, BAMET−UD2, and BAMET−R2, as well as for the dual receptor agonist INT−767 (176–179). Future studies should focus on elucidating the structure−activity relationships that govern their tissue specificity and immunomodulatory effects to accelerate the translation of engineered BA derivatives into clinically viable anticancer agents.

## 6 Immune–metabolic crosstalk: a dynamic regulatory network shaping disease pathogenesis

The crosstalk between immunity and metabolism constitutes a complex and dynamic regulatory network, in which metabolites serve not only as substrates for cellular energy and biosynthesis but also as critical signaling molecules that profoundly shape the differentiation, function, and fate of immune cells (180). The core of this framework is that various metabolites, including glucose, lipids, amino acids, and BAs, precisely regulate innate and adaptive immune responses through specific receptors and signaling pathways.

### 6.1 BAs as metabolic regulators of immune cell function

BAs serve as crucial signaling molecules that bridge microbial metabolism with host immunity, shaping immune cell differentiation, function, and inflammatory phenotypes through metabolic reprogramming. At the metabolic level, BAs can remodel mitochondrial function and redox balance. For instance, isoalloLCA promotes Treg differentiation by inducing mitochondrial ROS to activate NR4A1, whereas CDCA directly impairs mitochondrial function, triggering lipid peroxidation and ferroptosis (8, 121). Conversely, alloLCA suppresses RORγt activity and downregulates key lipogenic (e.g., Srebf1 and Elovl4) and gluconeogenic genes (e.g., Gck and Pck), while fatty acid β−oxidation pathways upregulate, effectively attenuating the flux of pro−inflammatory and pro−fibrotic signals from adipose tissue to the liver (53). Furthermore, modulation of the intestinal BA profile by *Bacteroides uniformis* downregulates inflammation−related pathways (e.g., IL−17, NF−κB, and MAPK) and upregulates lipid−metabolic pathways, such as PPAR signaling (107). As central mediators of metabolism−immune crosstalk, BAs shaped by microbial modification and host receptor engagement precisely calibrate immune responses, offering novel targets for intervening in metabolic inflammation and tumor immunotherapy.

### 6.2 Immune-driven BAs dysregulation in autoimmune and inflammatory diseases

BAs dysregulation is a potent driver of metabolic dysfunction in various diseases. The intricate crosstalk between immune activation and BAs dysregulation is the core pathogenic mechanism across autoimmune, infectious, and neurological diseases. A core feature of autoimmune hepatitis is the reprogramming of BA metabolism, marked by elevated serum conjugated BAs levels and disrupted enterohepatic circulation, which directly fuels hepatic inflammation and offers diagnostic potential (181). Furthermore, inflammation, oxidative stress, BA metabolism disorders, and gut-liver axis dysregulation significantly increase the risk of NAFLD/metabolic dysfunction-associated fatty liver disease (MAFLD) (182).

## 7 Conclusion

BAs have emerged as versatile signaling molecules that critically shape immune responses in both physiological and pathological contexts. This review illustrates how BAs fine-tune the activation, differentiation, and function of diverse immune cells through a growing repertoire of receptors and in close interplay with the gut microbiota. Their roles range from maintaining intestinal homeostasis and liver immune balance to modulating neuroinflammation and editing the tumor microenvironment. However, the translation of BA-based interventions from preclinical models to human diseases remains challenging, primarily due to microbial modification, tissue-specific receptor expression, species-specific metabolic differences, context-dependent immune responses that are difficult to replicate, and insufficient systematic evaluation of potential side effects. Despite these hurdles, advancing our understanding of cell- and context-specific BA signaling offers exceptional opportunities for therapeutic innovations. Future efforts should prioritize the development of targeted receptor modulators, engineered microbial therapeutics, and personalized dietary strategies that can selectively redirect BA-driven immunity toward protective or antitumor outcomes. Integrating multi-omics profiling with functional immune assays is essential to decode the dynamic BA-immune crosstalk in health and disease, ultimately paving the way for novel, mechanism-based interventions in immunology.

## Glossary

BAs: Bile Acids
CA: Cholic Acid
CDCA: Chenodeoxycholic Acid
GCA: Glycocholic Acid
TCA: Taurocholic Acid
TCDCA: Taurochenodeoxycholic Acid
GCDCA: Glycochenodeoxycholic Acid
LCA: Lithocholic Acid
DCA: Deoxycholic Acid
TDCA: Taurodeoxycholic Acid
GDCA: Glycodeoxycholic Acid
UDCA: Ursodeoxycholic Acid
TUDCA: Tauroursodeoxycholic Acid
GUDCA: Glycoursodeoxycholic Acid
TLCA: Taurolithocholic Acid
GLCA: Glycolithocholic Acid
Tfh: T Follicular Helper
3-oxoDCA: 3-oxodeoxycholic Acid
3-oxoLCA: 3-oxolithocholic Acid
3−oxoalloLCA: 3−oxoallo−lithocholic acid
alloLCA: allo−lithocholic Acid
α-MCA: α-muricholic Acid
isoLCA: Isolithocholic Acid
HDCA: Hyodeoxycholic Acid
isoalloLCA: isoallolithocholic Acid
isoDCA: 3β-hydroxydeoxycholic Acid
ω-MCA: ω-muricholic Acid
NorUDCA: 24-Nor-ursodeoxycolic Acid
RORγT: Retinoic Acid Receptor-related Orphan Receptor-γT
TGR5: Takeda G protein-coupled receptor 5
CNS1: Conserved Noncoding Sequence 1
CNS3: Conserved Noncoding Sequence 3
VDR: Vitamin D Receptor
FXR: Farnesoid X Receptor
NR4A1: Orphan Nuclear Receptor 4A1
TGF-β: Transforming Growth Factor β
3-oxo-Δ^4,6^-LCA: 3-oxo-Δ^4,6^-lithocholic Acid
HCC: Hepatocellular Carcinoma
NK: Natural Killer
T-β-MCA: Tauro-β-muricholic Acid
CREB: Cyclic-AMP Response Element-Binding Protein
CXCR6: C-X-C Motif Chemokine Receptor 6
CXCL16: C-X-C Motif Chemokine Ligand 16
TNF-α: Tumor Necrosis Factor α
IFN-γ: Interferon-γ
Tfh: T Follicular Helper
RRMS: Relapsing-Remitting Multiple Sclerosis
IL-17: Interleukin-17A
IBD: Inflammatory Bowel Disease
UC: Ulcerative Colitis
CD: Crohn’s Disease
7-ketoLCA: 7-ketolithocholic acid
12-KLCA: 12-ketolithocholic acids
CRC: colorectal cancer
iNOS: inducible Nitric Oxide Synthase
RA: Rheumatoid Arthritis
AML: Acute Myeloid Leukemia
MAIT: Mucosa-associated Invariant T
CA7S: Cholic Acid 7-Sulfate
PSC: Primary Sclerosing Cholangitis
ILC: Innate Lymphoid Cell
GPCR: G protein-coupled receptors
6E-CDCA: 6-ethylchenodeoxycholic Acid
RORs: Retinoid Orphan Receptors
DCs: Dendritic cells
ILC3s: Group 3 Innate Lymphoid Cells
NKT: natural killer T
MCA: muricholic acid
IL−1β: Interleukin-1β
IL−6: Interleukin-6
NLRP3: NOD-like receptor pyrin domain containing 3
Treg: regulatory T cell
SHP: small heterodimer partner
CYP7A1: cholesterol 7α-hydroxylase
DSS: dextran sulfate sodium
FGF15: Fibroblast Growth Factor 15
CAR: Constitutive Androstane Receptor
hAR: human Androgen Receptor
LPS: lipopolysaccharide
JNK: c-Jun N-terminal kinase
NASH: Nonalcoholic Steatohepatitis
PBC: Primary Biliary Cholangitis
OCA: obeticholic acid
SXR: Xenobiotic Receptor
PXR: Pregnane X Receptor
LXR: Liver X receptor
BSHs: Bile Salt Hydrolases
HSDHs: Hydroxysteroid Hehydrogenases
FMT: Fecal Microbiota Transplantation
NSCLC: Non−small Cell Lung Cancer
CNS: Central Nervous System
ROS: Reactive Oxygen Species
PDAC: Pancreatic Ductal Adenocarcinoma
MAFLD: Metabolic Dysfunction-associated Fatty Liver Disease
Th17: T helper 17 cells
